# The Novel Cis-Encoded Small RNA h2cR Is a Negative Regulator of *hfq2* in *Burkholderia cenocepacia*

**DOI:** 10.1371/journal.pone.0047896

**Published:** 2012-10-17

**Authors:** Christian G. Ramos, Paulo J. P. da Costa, Gerd Döring, Jorge H. Leitão

**Affiliations:** 1 Institute for Biotechnology and Bioengineering, Instituto Superior Técnico, Universidade Técnica de Lisboa, Lisboa, Portugal; 2 Department of Bioengineering, Instituto Superior Técnico, Universidade Técnica de Lisboa, Lisboa, Portugal; 3 Institut für Medizinische Mikrobiologie und Hygiene, University of Tübingen, Tübingen, Germany; The Methodist Hospital Research Institute, United States of America

## Abstract

Small non-coding regulatory RNAs (sRNAs) post-transcriptionally affect multiple phenotypes in prokaryotes and eukaryotes, yet most of the underlying regulatory mechanisms and the nature of the target mRNAs remain unclear. Here we report the identification and functional analysis of the novel cis-encoded sRNA h2cR, from the human opportunistic pathogen *Burkholderia cenocepacia* J2315. The sRNA was found to negatively regulate the *hfq2* mRNA, through binding to part of the 5′-UTR region of the *hfq2* mRNA, resulting in accelerated *hfq2* mRNA decay and reduced protein levels in exponentially growing cells. Both the h2cR transcript and the *hfq2* mRNA are stabilized by the other *B. cenocepacia* RNA chaperone, Hfq. Infection experiments using the nematode *Caenorhabditis elegans* revealed that down-regulation of Hfq2 by h2cR decreases the *B. cenocepacia* ability to colonize and persist within the nematode, suggesting a role for h2cR on bacterial persistence in the host.

## Introduction

Small RNAs (sRNAs) post-transcriptionally regulate multiple phenotypic traits in prokaryotes and eukaryotes. In bacteria, sRNAs have been implicated in the response to environmental stress [1], [2] and, more recently, in the regulation of virulence [3]–[8]. Bacterial sRNAs can be classified as cis-encoded sRNAs, with extended complementarity to their target mRNA and located in the same DNA region, and trans-encoded sRNAs which only partially hybridize with their mRNA target, being encoded in distinct *loci* from it [9]. Most of the known bacterial sRNAs require the action of the RNA chaperone Hfq to promote interaction with their targets [10]. The Hfq RNA chaperone has been shown to facilitate sRNAs/mRNAs transactions by stimulating RNA duplex formation between sRNAs and their mRNA targets [11]–[13]. Although most of the known Hfq proteins are required sRNAs/mRNAs interactions, in the case of *S. aureus*, sRNA/mRNA interactions seem to proceed without the requirement of Hfq [14]. However, *hfq* regulation itself is only incompletely understood, particularly in organisms which contain more than one Hfq protein, as it is the case of bacteria of the *Burkholderia cepacia* complex, composed of opportunistic human pathogens [15]–[18].

The soil bacterium *Burkholderia cenocepacia* harbors two distinct Hfq-like proteins, Hfq and Hfq2 [19], which are differentially expressed during cell growth: *hfq* mRNA reaches maximal levels in the early exponential growth phase, while *hfq2* mRNA is maximally expressed in the stationary phase [20]. Factor(s) involved in quorum sensing in *B. cenocepacia* have been implicated in the up-regulation of Hfq2 during the stationary phase [20]. Here we report on the identification of the novel sRNA h2cR which is actively transcribed during exponential growth of *B. cenocepacia* J2315. Data presented indicate that h2cR negatively regulates *hfq2* mRNA by interacting with its 5′-UTR. Results obtained upon silencing h2cR in *B. cenocepacia* J2315 indicate a reduced ability of the opportunistic pathogen to colonize the *Caenorhabditis elegans* used as a infection model, implicating this sRNA in the persistence of the bacterium at least in this particular host.

## Materials and Methods

### Bacterial strains and culture conditions

*B. cenocepacia* strains used in this work (Table 1) were maintained on PIA plates (Pseudomonas Isolation Agar, Becton Dickinson, New Jersey, USA), supplemented with 850 µg ml^−1^ trimethoprim or 350 µg ml^−1^ chloramphenicol or kanamycin, when appropriate. LB medium, supplemented with antibiotics when appropriate, was used for liquid cultures which were orbitally agitated (250 rev min^−1^, 37°C) using an Agitorb 200 incubator (Aralab, Portugal).

**Table 1:** Bacterial strains and plasmids used in this work.

Strain	Description	Reference or source
*B. cenocepacia* J2315	CF sputum isolate	[44]
*B. cenocepacia* CJ1	*B. cenocepacia* J2315 derivative with the *hfq* gene interrupted by a Tp gene cassette	[20]
*B. cenocepacia* CJ2	*B. cenocepacia* J2315 derivative with the *hfq2* gene interrupted by a non-sense stop mutation	[20]
*B. cenocepacia* J2315+ pSAS3	*B. cenocepacia* J2315 harbouring plasmid pSAS3	[20]
*B. cenocepacia* CJ1+ pSAS3	*B. cenocepacia* CJ1 mutant harbouring plasmid pSAS3	[20]
*B. cenocepacia* CJ4	*B. cenocepacia* J2315 harbouring plasmid pCGR17	This study
*B. cenocepacia* CJ5	*B. cenocepacia* J2315 harbouring plasmid pCGR18	This study
*B. cenocepacia* CJ6	*B. cenocepacia* CJ2 mutant harbouring plasmid pCGR27	This study
*B. cenocepacia* CJ7	*B. cenocepacia* CJ4 harbouring plasmid pCGR27	This study
*B. cenocepacia* CJ8	*B. cenocepacia* CJ5 harbouring plasmid pCGR27	This study
*B. cenocepacia* CJ9	*B. cenocepacia* J2315 harbouring plasmid pCGR31	This study
*B. cenocepacia* CJ10	*B. cenocepacia* J2315 harbouring plasmid pCGR32	This study
*B. cenocepacia* CJ11	*B. cenocepacia* J2315 harbouring plasmid pMLBAD	This study
*B. cenocepacia* CJ12	*B. cenocepacia* J2315 harbouring plasmid pBBR1	This study
*E. coli* BL21	Host for recombinant protein expression	Stratagene
*E. coli* TOP10 F'	Host for recombinant plasmids	Invitrogen
Plasmids		
pCR II	Ap^R^; Km^R^; TOPO-TA cloning plasmid	Invitrogen
pET23a+	Ap^R^, used for C-terminal 6×histidine-tagged protein expression.	Stratagene
pMLBAD	Tmp^R^; used for gene expression in Bcc	[45]
pBBR1MCS	Cm^R^; used for gene expression in Bcc	[46]
pSAS5	pWH884 with the *hfq2* gene cloned	[20]
pSAS6	pET23a+ with the *ompA* gene clone	(Sousa, SA, Leitão JH) Unpublished data
pSAS3 (p*hfq*)	pMLBAD with the *hfq* gene cloned	[21]
pCGR4	pET23a+ with the *hfq* gene	[21]
pCGR10	pCR II with the 136 bp cDNA fragment of the h2cR sRNA TA-cloned	This study
pCGR11	pCR II with the 668 bp cDNA fragment of the *hfq2* mRNA TA-cloned	This study
pCGR12	pCR II with the 216 bp cDNA fragment of the 5′UTR *hfq2* RNA TA-cloned	This study
pCGR13	pCR II with the 216 bp cDNA fragment of the5′UTR *hfq2* RNA cloned in the HindIII/BamHI sites (T7 promoter control)	This study
pCGR14	pCR II with the 136 bp cDNA fragment of the h2cR RNA cloned in the HindIII/BamHI sites (T7 promoter control)	This study
pCGR17 (ph2cR)	pMLBAD with the 136 bp cDNA fragment of the h2cR RNA cloned in the XbaI/HindIII sites	This study
pCGR18 (h2cR^sil^)	pMLBAD with the 104 bp anti sense cDNA fragment of the h2cR with a cca extension cloned in the EcoRI/XbaI sites	This study
pCGR26	pCGR13 with the his-tagged Hfq2 cloned in the HindIII/EcoRI sites	This study
pCGR27 (pThfq2)	pBBR1MCS with the N-terminus 6xhistidine tagged *hfq2* and its complete 5′UTR cloned in the XhoI/HindIII sites	This study
pCGR28	pCR II with the 180 bp cDNA fragment of the −220 to −40 region of *hfq2* 5′UTR cloned in the XbaI/HindIII sites	This study
pCGR29	pCR II with the 100 bp cDNA fragment of the −70 to +30 region of *hfq2* 5′UTR cloned in the XhoI/BamHI sites	This study
pCGR30	pCR II with the250 bp cDNA fragment of the −220 to +30 region of *hfq2* 5′UTR cloned in the XbaI/NotI sites	This study
pCGR31	pBBR1 with the 136 bp cDNA fragment of the h2cR RNA cloned in the XbaI/HindIII sites	This study
pCGR32	pBBR1 with the 104 bp anti sense cDNA fragment of the h2cR with a cca extension cloned in the EcoRI/XbaI sites	This study

### DNA manipulation techniques

Total DNA was obtained from exponentially growing cells of *B. cenocepacia* J2315 using the High pure PCR template preparation kit (Roche, Indianapolis, USA). Plasmids used in this work are described in Table 1 and oligonucleotides are listed in Table 2. Amplification of *B. cenocepacia* J2315 *hfq2, hfq2* 5′-UTR, and h2cR was achieved with adequate primers (Table 2). PCR products were purified from 0.8 % agarose gels, after electrophoresis, using the NucleoExtract II kit (Machery-Nagel, Hannover, Germany), and were used for plasmid construction either by TA-cloning of the DNA fragments or by ligation of endonuclease-digested PCR fragments to the corresponding linearized plasmid. Resulting plasmids were used in PCR experiments with adequate primer combinations (M13 reverse primer and the appropriate upper primer) to confirm the correct orientation of the cloned DNA fragment. Plasmids pCGR11 (*hfq2* mRNA), pCGR13 (*hfq2* 5′-UTR), pCGR14 (h2cR sRNA), pCGR28 (nt −220 to −40 of *hfq2* 5′-UTR), pCGR29 (nt −70 to +30 of *hfq2* 5′-UTR) and pCGR30 (nt −220 to +30 of *hfq2* 5′-UTR) were used for *in vitro* transcription experiments. Silencing of the h2cR sRNA was achieved by expressing a truncated (104 nt) anti-sense h2cR RNA derivative, containing a 3′ proximal CCA extension. Plasmid pCGR26 was constructed by cloning the HindIII/EcoRI fragment of plasmid pSAS5 [20] containing the *hfq2* gene fused with a six-histidine tag at the N-terminus, into the HindIII/EcoRI sites of pCGR13. Plasmid pCGR27 was constructed by cloning the fragment obtained from restriction of pCGR26 with XhoI/HindIII into the XhoI/HindIII sites of pBBR1MCS, allowing the expression and translation of the His-tagged Hfq2 from its own promoters. pBBR1MCS and derivatives are fully compatible with pMLBAD [18], [19]. Plasmid pCGR31 (+h2cR) was constructed by sub-cloning the XbaI-HindIII fragment obtained from pCGR14 into the XbaI-HindIII sites of pBBR1MCS, while pCGR32 (h2cR^sil^) was constructed by cloning the EcoRI-XbaI fragment obtained from pCGR14 into the EcoRI-XbaI sites of pBBR1MCS. These plasmids allow the constitutive expression of the cloned genes in *B. cenocepacia* (Ramos, da Costa and Leitão, unpublished results). All plasmid constructions were confirmed by DNA sequencing.

**Table 2:** Oligonucleotides and primers used in this work.

Name	Sequence (5′–3′)	Purpose	Reference or source
M13 Fwd M13 Rev	CTGGCCGTCGTTTTAC CAGGAAACAGCTATGAC	PCR screening of clones and transcription template Northern analysis of *hfq* Northern analysis of *hfq2* Northern analysis of 5S rRNA	Invitrogen Invitrogen [20]
HFQ	AAAGGGCAATTGTTACAAG		
HFQ2	TACGGCTCCCGCGAGCCGCGTGAA		[20]
5S	TTCGGGATGGGAAGGGGTGGGA		[20]
NBh2c	TTACGGTTCGACACAATCAC	Northern blot analysis of h2cR	This study
T7-TSS-H2	ATACGGGGCTGCGAGTCGT	In vitro transcription of the of *hfq2* mRNA In vitro transcription of the of *hfq2* mRNA	This study
Hfq2c_Rev	TTGTCGACTTACTGGCCGTCCGGCAC		[20]
5H2-H	TTAAGCTTGCCTCTTGTTCATGCTCC	In vitro transcription of the 5′UTR of *hfq2 *In vitro transcription of the 5′UTR of *hfq2*	This study
5H2-B	TTGGATCCTGAGGTGCGGCAGGAGCC		This study
h2cR-H	TTTTAAGCTGCGTCAGAGAAT	In vitro transcription of the h2cR sRNA In vitro transcription of the h2cR sRNA	This study
h2cR-B	TTGGATCCGCGAAAATGCGCC		This study
h2cR-X	TTTCTAGAGTGCGTCAGAGAAT	Overexpression of h2cR	This study
h2cR-H2	TTAAGCTTGCGAAAATGCGCC	Overexpression of h2cR	This study
h2cR-E	TTGAATTCTGCGTCAGAGAAT	Silencing of h2cR	This study
h2cR-Xcca	TTCTAGAGTTCAAAATGCGCC	Silencing of h2cR	This study
CGRO100	TTTTCTAGACCGTTCTATATTGACGG	Amplification of the −220 region	This study
CGRO101	TTAAGCTTTTGCTGAAGTTGTTCGGT	Amplification of the −40 region	This study
CGRO103	TTCTCGAGTGATTGTGTCGAACCGTA	Amplification of the −70 region	This study
CGRO104	TTGGATCCTGCGGATGGGATTCTGCGGG	Amplification of the +30 region	This study
5′ RACE Adapter	5′-GCUGAUGGCGAUGAAUGAACACUGC GUUUGCUGGCUUUGAUGAAA-3′	5′ RACE h2cR	Ambion
5′ RACE Outer Primer	5′-GCTGATGGCGATGAATGAACACTG-3′	5′ RACE h2cR	Ambion
5′ RACE Inner Primer	5′-CGCGGATCCGAACACTGCGTTTGCTGGCTTTGATG-3′	5′ RACE h2cR	Ambion
5′R	5′-AATCACGACTGCGCGCTCCC-3′	5′ RACE h2cR	This study

### Northern blot analysis

The levels of transcripts corresponding to *hfq2* mRNA or h2cR sRNA were assessed by Northern blot analysis using 2 µg of total RNA purified from cells of *B. cenocepacia* J2315 and derivative strains. Total RNA was loaded into 6 or 15 % and 8 M urea polyacrylamide gels for *hfq2* and h2cR respectively, and electrophoresed in 1× TBE buffer at 35 mA. After electrophoresis, total RNA was electrotransferred at 20 V, for 16 h at 4°C using 0.5× TBE buffer to a BrightStar Plus membrane (Ambion, Madrid, Spain). Oligonucleotide probes (Table 2) were labeled with the BrightStar Biodetect kit (Ambion) and purified using Quick-Spin columns (Roche). Pre-hybridization and hybridization procedures were based on previously described methods [20]. The 5S rRNA was used as control in all Northern blot analysis experiments. Hybridization signals were detected using the BrightStar Biodetect kit (Ambion) and Kodak MX X-ray films. Relative expression analysis was estimated using the ImageJ software and the band intensities of the 5S rRNA as standard.

### RNA levels and decay experiments

Total RNA was obtained from cells of *B. cenocepacia* J2315 and derivative strains using the Ribopure RNA isolation kit (Ambion) or the mirVana miRNA isolation kit (Ambion), for total RNA or sRNA-enriched fractions, respectively. RNA concentration was estimated in a ND 1000 spectrophotometer (Nanodrop). RNA labeling with biotin was conducted as previously described [20], [21]. In RNA decay experiments, 500 µg.mL^−1^ of rifampicin were added, immediately before the assay, to cell cultures at 24 h of growth for *hfq2* assays or at 2 h, 8 h or 24 h for h2cR assays. 2 µg of total RNA were used in each experiment. Films were scanned and the intensity of RNA bands was measured with the ImageJ software. The intensities were plotted in semi-log plots and RNA half-life was calculated using the slope of each plot.

### Electrophoretic mobility shift (EMSA) assays

EMSA assays with the h2cR transcript, the *hfq2* coding region, the *hfq2* full mRNA and the 5′-UTR of the *hfq2* transcript and derivatives were performed as previously described [20]. The His-tagged Hfq protein used in EMSA assays was prepared as previously described [21]. Briefly, for EMSA assays, 2.5 nM of the h2cR, together with 0, 0.1, 0.5 or 2.5 nM of the *hfq2* full transcript or the *hfq2* coding sequence, or increasing amounts of the *hfq2* 5′UTR derivatives (0, 0.1, 0.5, 2.5 or 10 mM) were incubated in 25 µl of RNA binding buffer [21] for 30 min at 25°C. The ability of Hfq to bind h2cR (2.5 nM) or *hfq2* 5′-UTR (2.5 nM), was evaluated by EMSA assays using 0, 0.5, 1, 5 or 10 nM of the hexameric form of His-tagged Hfq (Hfq_6_), or 0, 0.5, 1, 5, 10 or 50 nM of His-tagged Hfq_6_, respectively. Non-labelled yeast tRNA (Ambion) was added in excess to each sample to minimize non-specific binding. Incubation, resolution of RNA-protein complexes and detection of band-shifts was performed as described before [21].

### RNA in vitro transcription and labeling

The cDNA templates for the *hfq2* mRNA full transcript, the *hfq2* 5′-UTR, the *hfq2* coding region (CDS), the nt −220 to −40 (Start), the nt −70 to +30 (Mid) and nt −220 to +30 (End) 5′-UTR, or the h2cR sRNA, were generated by PCR, using the following primer pairs and DNA templates: T7-TSS-hfq2– hfq2Low, and plasmid pCGR11; 5H2-H –5H2-B, and plasmid pCGR12; Up_hfq2_C – Low_hfq2_C, and plasmid pCGR8; CGRO100– CGRO101, and plasmid pCGR28; CGRO102– CGRO103, and plasmid pCGR29; CGRO100– CGRO103, and plasmid pCGR30, or h2cR-X – h2cR-H2, and plasmid pCGR14. All RNA transcripts were generated from the T7 promoter, using the MEGAshortscript kit (Ambion). The transcripts h2cR (136 nt), 5′-UTR (216 nt), Start (180 nt), Mid (100 nt), and End (250 nt) were purified from 8%−7 M urea polyacrylamide gels, while the *hfq2* derivatives CDS (585 nt) and the full transcript (801 nt) where purified from a 1% TBE/agarose gel. RNAs were subsequently ethanol-precipitated and 5′ end-labelled with 11-UTP-biotin (Ambion). RNA 5′end-labelling was achieved after dephosphorylation with 2 U of calf intestinal phosphatase (New England Biolabs, NEB, Ipswich, USA) for 15 min in NEB buffer 2, with T4 polynucleotide kinase (PNK, NEB). Briefly, 200 pmol of RNA were mixed with 7.5 nmol of 11-UTP-Biotin, in a total volume of 50 µl, containing 1 mM ATP, 5 µl of 10× T4 PNK buffer, and 10 U of T4 PNK. Reactions were carried out at 37°C for 30 min. RNA was phenol-chloroform extracted, ethanol precipitated, and finally cleaned-up with Ilustra NAP-5 columns (GE Healthcare, Pollards Wood, UK).

### Rapid amplification of 5′ ends of cDNA (5′RACE)

Amplification of the cDNA end corresponding to the h2cR sRNA was achieved by using total RNA purified from *B. cenocepacia* J2315 cells in the early exponential phase (2 h), exponential phase (8 h) or in the stationary phase (24 h). RNA was isolated from cells using the mirVana kit (Ambion) for h2cR, followed by DNase I treatment with TurboDNase (Ambion). Amplification of the 5′-end of cDNA was carried out using the First Choice RLM Race kit (Ambion), using 20 pmol of the primer 5′R (5′RACE of h2cR) (Table 2) and 5 µg of total RNA. Amplification products were fractionated in 1% agarose gels and 7.5% PAGE, and visualized after Sybr Gold Staining (Invitrogen Madrid, Spain). Amplification fragments were purified from the gel with the Nucleospin II kit (Macherey-Nagel), and used for cloning into plasmid pCR II-TOPO. Resulting plasmids were used to transform *E. coli* Top10 F'. Plasmids from selected clones were sequenced.

### Determination of Hfq2 levels

The effect of the h2cR sRNA on the levels of the Hfq2 protein was assessed by western blotting. For this purpose, plasmid pCGR27 (pThfq2), which is able to drive the transcription of *hfq2* fused with a nucleotide sequence encoding a six histidine tag at the N-terminus from each of the *hfq2* own promoters, was introduced into *B. cenocepacia* CJ2 (Δ*hfq2*), yielding *B. cenocepacia* CJ6. The CJ6 strain was further transformed with plasmids pCGR17 or pCGR18, which allow the h2cR overexpression (strain CJ8) or silencing (strain CJ7), respectively. The cultures were grown for 24 h in liquid LB, supplemented with 650 µg ml^−1^ trimethoprim and 1% L-arabinose. Culture aliquots of 1 ml were taken, the protein was purified with the Illustra TriplePrep kit (GE Healthcare), and ressuspended in 100 µl of 2D-DIGE buffer (30 mM Tris-HCl, 4% CHAPS, 2 M thiourea, 7 M urea, pH 8.5). Protein concentration was estimated in a Nanodrop 1000 spectrophotometer. Aliquots containing 20 µg of total protein were separated by 12% SDS-PAGE and then electroblotted onto a nitrocellulose membrane, using Towbin transfer buffer, for 45 min at 120 mA. The 6xHis-tagged Hfq2 protein was detected using the PentaHis-HRP antibody system (Qiagen Madrid, Spain) with tetramethylbenzidine (Sigma, St. Louis, USA) as substrate.

### Nematode infection experiments

Nematode killing assays and bacterial colonization of the digestive tract of the nematodes were performed based on previously described methods [21], using the *C. elegans* mutant strain DH26, cultured in NGM II medium, supplemented with 2% L-arabinose, when appropriate. Total protein and RNA were also obtained from *B. cenocepacia* strains CJ6, CJ7 and CJ8, when infecting the nematode *C. elegans*. Worms were lysed using silica-carbide beads (Biospec Products Inc, USA), based on previously described methods [22]. Total bacterial RNA was obtained from total RNA after purification with the Microbe-Enrich kit (Ambion), following the manufacturer instructions. After clean-up of the bacterial total RNA with the mirVana kit (Ambion), aliquots were used for Northern blot analysis experiments to assess the levels of the *hfq2* mRNA, the h2cR transcript, and the 5S rRNA. Total protein was obtained with the Illustra TriplePrep kit (GE Healthcare), and processed as described above. Protein aliquots were used to assess the Hfq2 levels by western blot, as described above.

### Bioinformatics

BLAST searches were performed using the Integrated Microbial Genomes (IMG) webserver (E-value ≤ 1e^−50^). The h2cR sRNA secondary structure was predicted using the Mfold algorithm [23], and modeled with the RNA structure program (v5.03). Alignment of h2cR and the 5′-UTR of *hfq2* was achieved using the RNAHybrid software [24]. Putative promoter were predicted using the Virtual Footprint and PRODORIC web tool [25].

### Statistics

An unpaired two-tailed-*chi* test was used to calculate the *P* values for the Western blotting experiments and the *C. elegans* assays. Analysis of data from Northern blotting was performed using a paired one-tailed *t* test to calculate the *P* values (***, *P*<0.001; **, *P*<0.005; *, *P*<0.01). Error bars represent the means of the standard deviation. Variance was computed based on ANOVA analysis. Images shown are representative of each the experiments repetitions performed. All experiments were repeated independently at least 4 times, using triplicates, with a minimum of n = 12.

## Results

### Identification and cloning of h2cR

**Figure 1:**
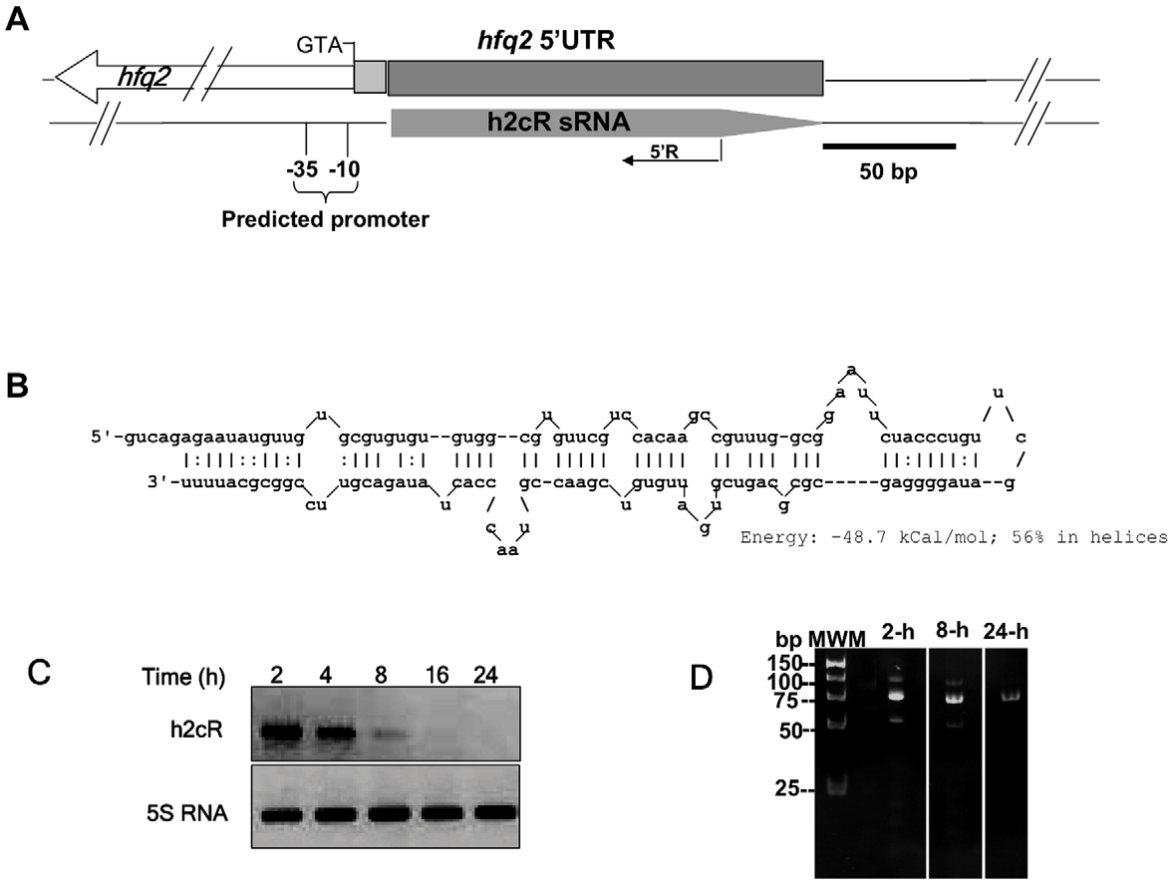
Genome location and transcriptional analysis of the h2cR sRNA in *B. cenocepacia* J2315. (A) Genomic location of h2cR, showing in the upper part the 5′-UTR of the*hfq2* gene, highlighting the direction of transcription of this gene (depicted by an open arrow and showing the ATG codon), the region where h2cR is inscribed (dark grey box), and the 5′-UTR region of *hfq2* lacking h2cR complementarity (light grey box). A solid arrow in the lower part represents the h2cR transcript. The oligonucleotide used for 5′-RACE (5′R) experiments with h2cR is also shown. (B) h2cR RNA sequence and predicted secondary structure, showing the predicted folding energy. (C) Northern blot analysis of h2cR transcription in *B. cenocepacia* J2315 cells harvested from cultures grown for the indicated time. (D) Photograph of a 7.5% PAGE gel after electrophoresis of the PCR products obtained from 5′-RACE experiments of the h2cR sRNA, using the specific oligonucleotide 5′R, and 1 µg of total RNA obtained from *B. cenocepacia* J2315 cells in the exponential (2 h), late-exponential (8 h) of stationary (24 h) phases of growth.

To identify sRNAs from *B. cenocepacia* which may interact with the RNA chaperone Hfq, we have co-purified the His-tagged Hfq protein together with *B. cenocepacia* sRNA fractions (<200 nt) [20], [21], followed by sequence analysis of the cloned cDNA fragments. This experimental approach allowed the identification of a novel sRNA, named h2cR (Hfq2 cis regulatory RNA), which was cloned into the pCR II cloning vector, yielding pCGR10. In silico analysis of *B. cenocepacia* J2315 chromosome 1 revealed that the h2cR encoding sequence ranged from nt 1704623 to 1704759 in the reverse strand of the 5′-untranslated region (UTR) sequence of *hfq2* (Fig. 1A). A putative promoter sequence upstream of the h2cR predicted transcription start site was bioinformatically identified (Fig. 1A), although the predicted −35 (gaTTGAGAgc) and −10 (aaTTAATAAcg) boxes consensus sequences found were highly degenerated. The h2cR secondary structure was predicted to be composed of a duplex, with an energy of −48.7 kCal/mol, with a 5′ overhang of 5 nucleotides (5′-gucag) and a 3′-terminal poly-U (Fig. 1B).

Bioinformatic analyses revealed the presence of h2cR homologues among all members of the Bcc, with an identity of at least 85%. h2cR homologues were also found among other *Burkholderia* species, with an identity of at least 75%. Remarkably, no h2cR homologues were found in other bacterial genomes (data not shown).

In order to analyze the pattern of h2cR transcript accumulation in cells at different growth phases, cultures of the *B. cenocepacia* J2315 (wt) strain were carried out in liquid LB at 37°C, and total RNA was extracted and used in Northern blot experiments. Results obtained showed that h2cR transcripts were maximal in cells at the early exponential phase of growth, decreasing to undetectable levels in cells at the stationary phase (Fig. 1C) in a pattern opposite to that previously described for the *hfq2* mRNA [18]. To determine the transcription start and stop sites of the h2cR sRNA, we have performed 5′-RACE experiments, followed by cloning and sequencing of the resulting cDNA amplimers. A major band of ∼75 bp was obtained with RNA samples from cultures grown for 2 h, 8 h or 24 h (Fig. 1D). Sequence determination allowed us to locate the h2cR Transcription Start Site (TSS) at nucleotide position 1704623 in *B. cenocepacia* J2315 chromosome 1.

### h2cR affects *hfq2* mRNA levels

**Figure 2:**
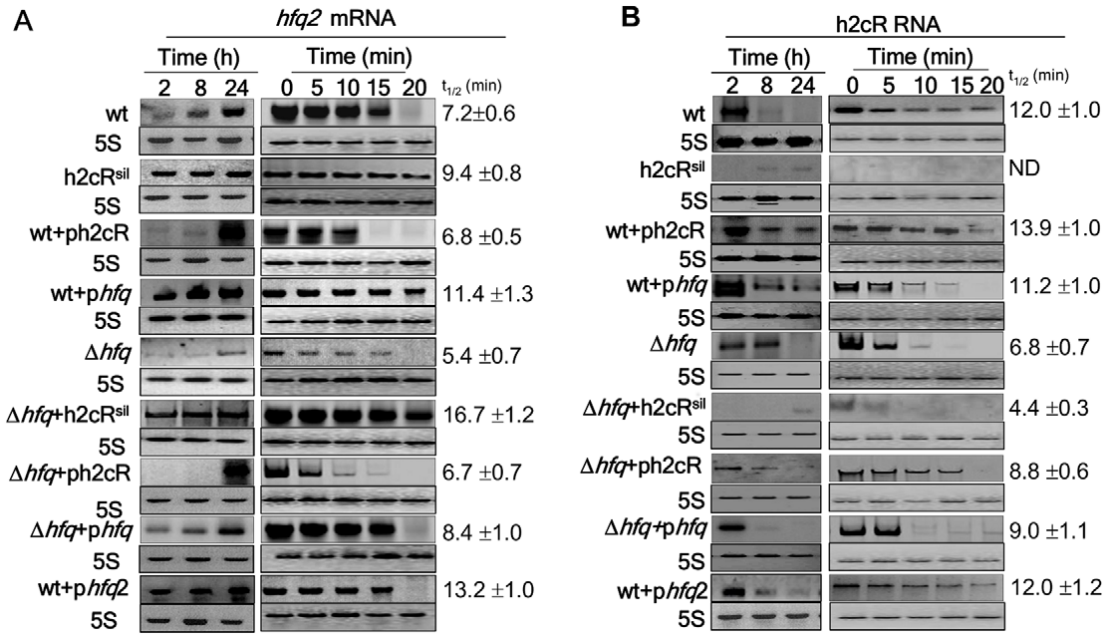
h2cR regulates *hfq2* mRNA levels. Northern blot analysis of the effects of h2cR overexpression (+ph2cR) or silencing (h2cR^sil^) on (A) *hfq2* mRNA levels (left panel) and *hfq2* mRNA stability (right panel) or (B) h2cR sRNA levels (left panel) and h2cR sRNA stability (right panel) in the wt, the Δ*hfq* mutant, and derivative strains. h2cR silencing or overexpression was achieved by means of expressing from a pBAD inducible plasmid a 104 nt truncated form of an h2cR-complementary RNA with a CCA trinucleotide extension, or the h2cR sRNA, respectively. The *hfq* mutant strain (Δ*hfq*) and derivative strains overexpressing *hfq* (Δ*hfq* +p*hfq*) or *hfq2* (Δ*hfq*+p*hfq2*) from a pBAD inducible plasmid [45] were also used to access the possible roles of Hfq, or Hfq2 on *hfq2* mRNA and h2cR expression and stability, respectively. RNA decay experiments were conducted using total RNA obtained from cells grown for 2-h (h2cR) or 24-h *(hfq2* mRNA). After addition of rifampicin (time 0), samples were taken at the indicated time. Half-life times (t_1/2_) were calculated by least-square fitting to the linear part of the logarithm of the percentage of remaining RNA versus time. Values indicated are the means of three independent experiments.

Since most known cis-encoded sRNAs exert their regulatory action on adjacent genes [9], *hfq2* was investigated as a possible mRNA target of h2cR, by means of over-expressing or silencing the sRNA. For this purpose, the wt strain was transformed with plasmid pCGR18, expressing a 104-nt anti-sense RNA that silences h2cR upon induction with arabinose. The resulting strain, CJ5, is hereafter referred as h2cR^sil^. The wt strain was also transformed with plasmid pCGR17 which overexpresses h2cR upon arabinose induction (wt+ph2cR). We have also included in these analyses a *B. cenocepacia* J2315 *hfq* mutant (strain CJ1) [20] and derivatives which either overexpress or silence h2cR, to assess any possible role played by Hfq on h2cR levels and/or stability. In the wt strain, as well as in the *hfq* mutant (Δ*hfq*), *hfq2* mRNA levels increased over time, reaching maximal values in cells at the stationary phase, with *hfq2* mRNA levels being higher in the wt strain than in the Δ*hfq* strain (Fig. 2A). Overexpression of h2cR in both the wt (wt+ph2cR) and the Δ*hfq* (Δ*hfq*+ph2cR) strains led to almost undetectable levels of *hfq2* mRNA during the exponential growth phase and high *hfq2* mRNA levels in cells at the stationary phase. In contrast, the silencing of h2cR led to higher *hfq2* mRNA levels during exponential phase in both the wt (h2cR^sil^) and the Δ*hfq* (Δ*hfq*+h2cR^sil^) strains (Fig. 2A). Interestingly, overexpression of *hfq* led to increased amounts of *hfq2* mRNA in both strains (wt+p*hfq* and Δ*hfq*+p*hfq*) during the exponential phase of growth, suggesting a possible role for Hfq in *hfq2* mRNA expression/stability. The results obtained when assessing the half-life of the *hfq2* mRNA on the different genetic backgrounds indicate that silencing of h2cR in the wt strain increased the *hfq2* mRNA half-life time to ∼30%, while overexpression of h2cR had almost no impact on the *hfq2* mRNA half-life time (reduction of only ∼6%) (Fig. 2A right panel). Interestingly, the *hfq2* mRNA half-life was also reduced in the *hfq* mutant, by approximately 25% (Fig. 2A). Taken together, these results indicate that h2cR negatively affects *hfq2* mRNA levels. In addition, results also suggest that Hfq plays a role in the stability of the h2cR transcript and the *hfq2* mRNA.

We have also assessed the levels of h2cR in the same strains used to assess the *hfq2* mRNA levels. The h2cR transcript levels were maximal in the early exponential growth phase in both the wt and the Δ*hfq* strains (Fig. 2B). In both strains, silencing of h2cR led to almost undetectable levels of h2cR transcripts in the exponential phase and to only barely detectable h2cR levels in the stationary phase. Contrasting with the observed h2cR transcripts accumulation throughout the exponential and stationary phase in the wt strain overexpressing h2cR, the corresponding RNA levels were surprisingly low in the *hfq* mutant strain overexpressing h2cR (Fig. 2B). Important differences were also observed in h2cR half-life. Values ranging from 11–14 min were estimated for h2cR in the wt strain and in the wt strain overexpressing h2cR, *hfq* or *hfq2* (Fig. 2B). However, in the *hfq* mutant and the *hfq* mutant overexpressing h2cR, the half-lives were reduced to 7–9 min. Taken together, our data strongly suggest that h2cR exerts a negative regulatory effect on *hfq2* mRNA, since higher h2cR levels correlate with lower or even absent levels of *hfq2* mRNA. A 43% and 25% reduction in h2cR sRNA and *hfq2* mRNA half-lives, respectively, were calculated for the Δ*hfq* strain, compared to the wt strain. Complementation of this mutation, using a *hfq* containing plasmid, partially restored the h2cR half-life to a value of ∼75% of that registered for the sRNA half-life in the wt strain, again suggesting that Hfq somehow stabilizes the h2cR sRNA.

### h2cR affects Hfq2 protein levels

**Figure 3:**
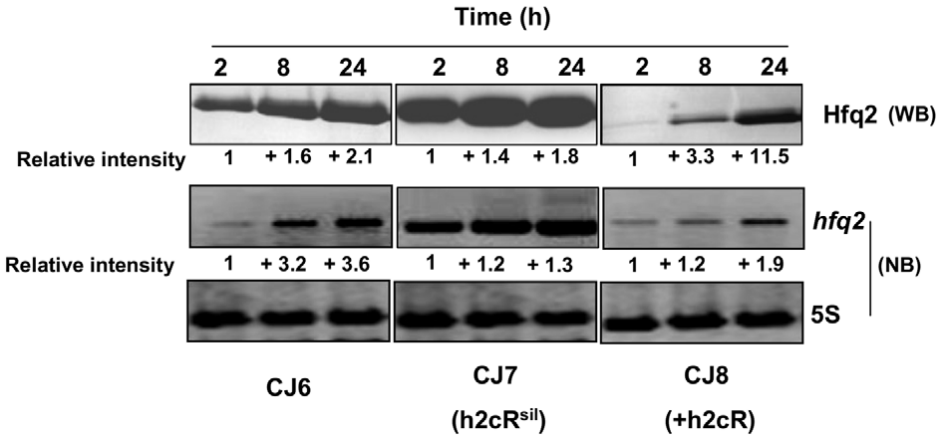
Analysis of the effect of h2cR overexpression or silencing on the Hfq2 protein and *hfq2* mRNA. Hfq2 levels were assessed by Western blotting (WB) while the *hfq2* mRNA levels were assessed by Northern blotting (NB). Transcription and translation experiments were performed using total RNA obtained from cells grown for the indicated time. The relative intensity of bands was estimated considering as unitary the intensity of the bands for cells of the CJ6, CJ7 or CJ8 strains, grown for 2 h, and using as reference the intensity of the 5S rRNA bands. Western blot analysis was performed using 20 µg of total protein per lane, estimated in a Nanodrop ND1000 spectrophotometer. Results shown are representative of at least 4 independent experiments.

The effects of h2cR silencing or overexpression on the levels of the Hfq2 protein produced were also assessed. For this purpose, the *B. cenocepacia* CJ2 mutant [20], in which the *hfq2* gene was deleted (Δ*hfq2*), was transformed with plasmid pCGR27 (expressing a 6 histidine-tagged *hfq2* mRNA from the *hfq2* own promoters), yielding strain CJ6. This strain allows the assessment of the levels of Hfq2 protein produced by Western blot using an antibody specific for the histidine-tag of the synthesized Hfq2 (see Materials and Methods). The CJ6 strain was further transformed with plasmids that allow h2cR overexpression or silencing, thus enabling the assessment of the effects of h2cR overexpression or silencing on the levels of the Hfq2 protein and the *hfq2* mRNA. CJ7 is a CJ6 derivative expressing an h2cR antisense transcript from plasmid pCGR18. CJ8 is a CJ6 derivative overexpressing h2cR from plasmid pCGR17. In the CJ6 strain, the levels of Hfq2 were lower in cells in the exponential phase of growth (2 and 4 h of growth), increasing to double the levels observed in cells at the stationary phase of growth (Fig. 3). The pattern of accumulation of *hfq2* mRNA in the CJ6 strain was similar to that observed for the protein Hfq2, although the levels increased by a 3.6 fold. For the CJ8 strain, the levels of the Hfq2 protein were barely detectable in cells after 2 h of growth and its levels increased by 11.5-fold in cells at grown for 24 hours (Fig. 3). Altogether, these results indicate that the levels of Hfq2 are negatively affected by h2cR, in good agreement with the already observed negative effect exerted by h2cR on the *hfq2* mRNA stability.

### h2cR regulates *hfq2* mRNA through binding within its 5′-UTR

**Figure 4:**
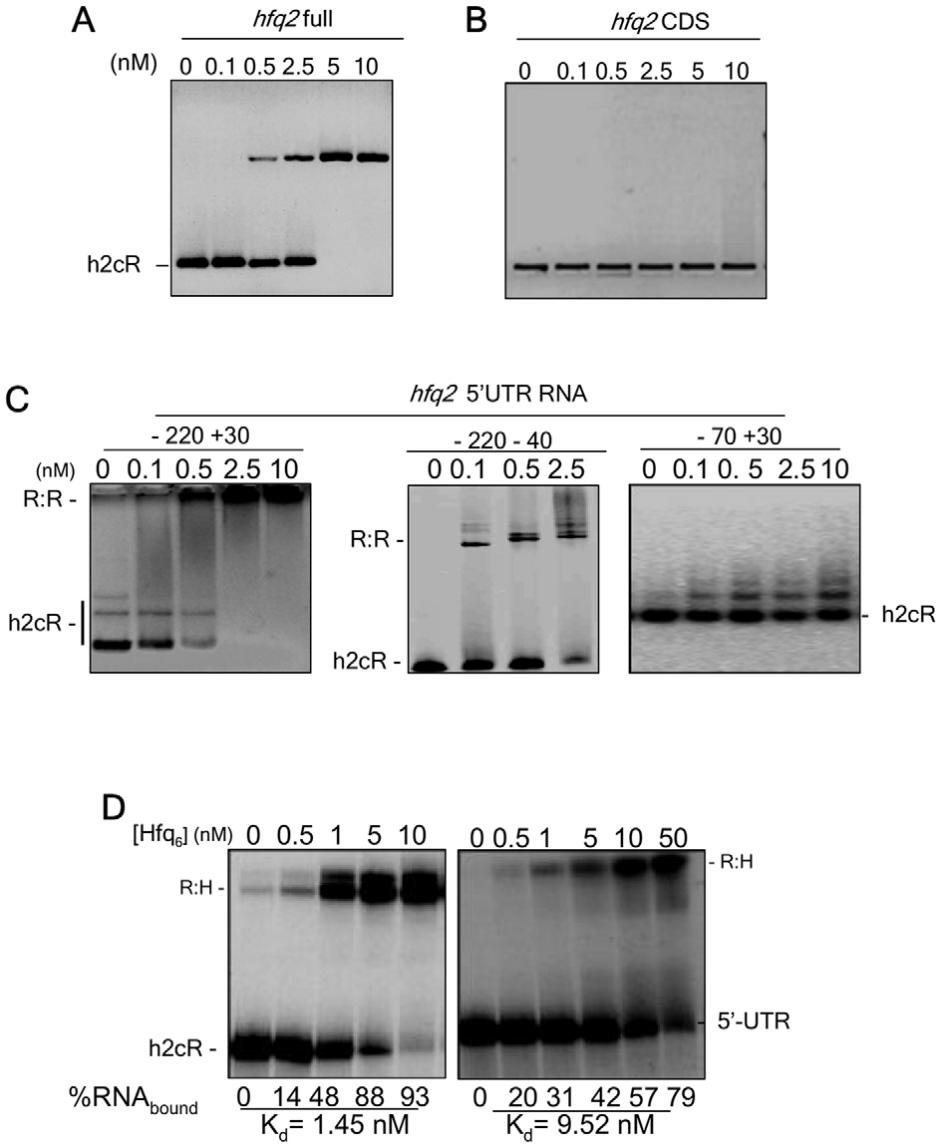
h2cR interacts specifically with the 5′UTR of *hfq2* mRNA. (A) EMSA experiments using 2.5 nM of the h2cR together with the indicated amounts of the *hfq2* full transcript or (B) the *hfq2* coding sequence; (C) EMSA experiments using with 2.5 nM of h2cR, together with the indicated amounts of the *hfq2* 5′UTR derivatives full 5′-UTR (nt −220 to +30), the RNA segment spanning nt −220 to −40 of the 5′-UTR, or the RNA segment spanning nt −70 to +30 of the 5′-UTR. For the full 5′-UTR and the RNA segment spanning nt −70 to +30 of the 5′-UTR 0, 0.1, 0.5, 2.5 or 10 nM of the respective 5′-UTR RNA derivatives were used, while for the RNA segment spanning nt −220 to −40 of the 5′-UTR 0, 0.1, 0,5, or 2.5 nM of RNA were used. (D) Ability of Hfq to bind 2.5 nM of h2cR (left panel) or the *hfq2* 5′-UTR (right panel), as evaluated by EMSA assays using the indicated concentrations of the hexameric form of Hfq. Apparent Kd values were calculated based on a semi-log plot of the RNA binding ratio versus protein concentration, using a exponential fit.

In order to gain further insights on the interaction of h2cR and the *hfq2* mRNA, we performed electrophoretic mobility shift assays (EMSA) to investigate the formation of complexes between the h2cR sRNA and the *hfq2* mRNA. Incubation of biotin-labeled h2cR with increasing concentrations of the complete *hfq2* mRNA (*hfq2* full, including the 5′-UTR) resulted in the formation of a slower migrating complex, consistent with an interaction between the two RNA molecules (Fig. 4A). In contrast, no complex was observed when the *hfq2* coding sequence (*hfq2* CDS) was incubated with the biotin-labeled h2cR, indicating that the 5′-UTR of *hfq2* is required for the RNA duplex formation (Fig. 4B). Therefore, additional EMSA assays were performed with the biotin-labelled h2cR sRNA, and three derivatives of the *hfq2* 5′-UTR: one RNA molecule spanning nt −220 to +30 (corresponding to the full 5′-UTR), a second RNA molecule spanning nt −220 to −40 (including most of the 5′-UTR), and a third RNA molecule spanning nt −70 to +30 (including the AUG region). h2cR formed RNA duplexes with the RNA molecule corresponding to the complete *hfq2* 5′-UTR (−220 +30) and with the RNA molecule spanning nt −220 to −40 of the *hfq2* 5′-UTR, but not with the *hfq2* mRNA derivative spanning the −70 to +30 of the *hfq2* 5′-UTR (Fig. 4C), indicating that h2cR and *hfq2* mRNA interactions are restricted to the nt region −220 to −40 of the *hfq2* 5′-UTR.

Next we sought to know whether Hfq forms complexes either with h2cR or with the *hfq2* mRNA. The results obtained indicate that Hfq interacts with a ∼6-fold higher binding affinity for h2cR (K_d_  = 1.5 nM), compared to the binding to the *hfq2* mRNA (K_d_  = 9.5 nM) (Fig. 4D). It is possible that the interaction of Hfq with each of these two RNA molecules contributes to their stabilization, in agreement with previous results showing reduced half-lives of both RNA molecules in an *hfq*-depleted genetic background (Fig. 2).

### Controlled levels of h2cR are required for the efficient colonization of *C. elegans*

Since sRNAs have been implicated in the regulation of virulence [3]–[8], we investigated the role of h2cR on the ability of *B. cenocepacia* J2315 to colonize and to kill the nematode *C. elegans*, previously used by our research group as an infection model [26]. Although extrapolations from non mammalian Bcc infection models to human infections should be used with caution [27], *C. elegans* has been successfully used to investigate the biology of a number of human pathogens [28].

**Figure 5:**
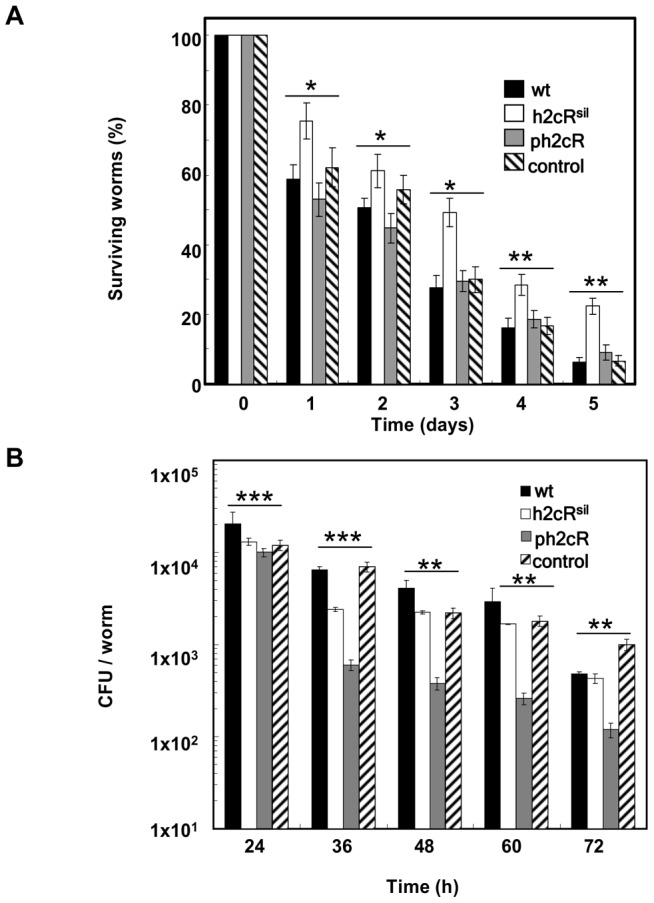
h2cR is required for *B. cenocepacia* J2315 virulence to *C. elegans*. (A) Ability of the *B. cenocepacia* strains J2315 (wt), either with the h2cR silenced (h2cR^sil^) or overexpressed (ph2cR), and the respective control plasmid (pBBR1MCS) to kill *C. elegans*. (B) Number of colony forming units (CFU) of *B. cenocepacia* strains J2315 (wt), either with the h2cR silenced (h2cR^sil^) or overexpressed (ph2cR) and the respective control plasmid (pMLBAD) colonizing the nematodes' digestive tract. The asterisks represent P values of the WT values versus all the rest.

With this objective, the silencing or over-expression of h2cR in the wt was achieved by transforming the strain with the plasmids pCGR18 or pCGR17, which express the h2cR anti-sense transcript of h2cR or overexpress the h2cR transcript upon induction with arabinose, respectively. Silencing of h2cR increased the survival of the nematodes, suggesting that h2cR affects *B. cenocepacia* virulence towards the nematodes (Fig. 5A). No significant differences in survival of the nematodes infected with the wt strain or the wt strain over-expressing h2cR were detectable (Fig. 5A). To check if the addition of arabinose by itself might have affected the results observed, we have cloned the h2cR coding sequence and the h2cR antisense sequence in plasmid pBBR1MCS, yielding plasmids pCGR31 and pCGR32, respectively. pBBR1MCS is leaky in *Burkholderia* strains and allows constitutive expression of the cloned genes from the *lac* promoter in the absence of IPTG induction (CG Ramos, PJ da Costa and JH Leitão, unpublished results). The *B. cenocepacia* wt, and the wt strain transformed with pCGR31 or pCGR32 were used in infection experiments similar to those described above. The results obtained were similar to those shown in Fig. 5A, indicating that the addition of arabinose did not influence the results obtained (data not shown).

We have also assessed the effects of h2cR overexpression or silencing on the total number of bacteria thriving inside the worm's digestive tract, using the wt strain and their derivatives transformed with pCGR17 or pCGR18. Silencing of the sRNA led to slight differences on the number of CFU per worm compared to controls (wt and wt transformed with pMLBAD), while h2cR overexpression led to a significant reduction of about one log in the total CFU per worm (Fig. 5B). Despite the reduction of the total CFUs per worm observed for the wt strain overexpressing h2cR, the ability of this strain to kill the nematodes was similar to that of the wt strain (Fig. 5B), suggesting the involvement of h2cR on bacterial survival inside the nematode's digestive tract, possibly by affecting the ability of the bacterium to adhere to the nematode's digestive tract. Previous work on the functional characterization of Hfq2 showed a more important role for this protein in killing the nematodes rather than in the colonization of their digestive tract [20], consistent with the effect here registered when silencing h2cR and thus promoting higher amounts of Hfq2. Furthermore, a *hfq2* null mutant still retained the ability to colonize and kill nematodes, a trait most probably derived from the expression of other Hfq- and/or Hfq2-independent virulence factors [20]. The results here presented suggest that the effects of h2cR on the persistence and killing ability of *B. cenocepacia* towards the nematode might result directly from the regulation exerted by the sRNA on the Hfq2. Nevertheless, our results also suggest that h2cR plays an additional role on the bacterium ability to kill the nematodes (Fig. 5A), besides the effects resulting from the regulation of the *hfq2* mRNA and, consequently, of the Hfq2 protein abundance.

### h2cR regulates *hfq2* mRNA levels in *B. cenocepacia* during infection

**Figure 6:**
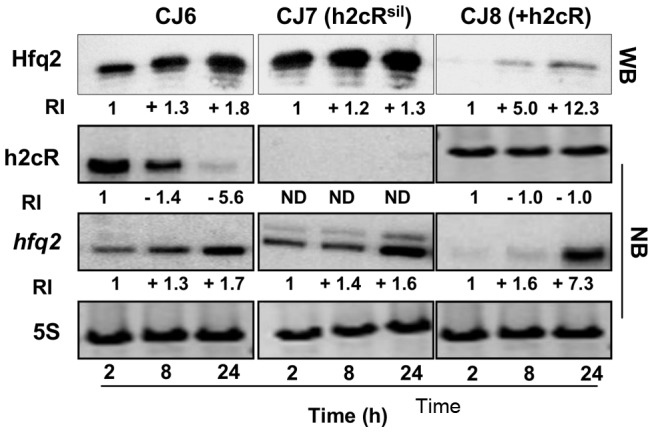
The h2cR sRNA affects the levels of the Hfq2 protein and the *hfq2* mRNA in *B. cenocepacia* cells infecting *C. elegans*. Levels of the Hfq2 protein and of h2cR transcript and *hfq2* mRNA in *B. cenocepacia* cells harvest from nematodes after the indicated post-infection time. Hfq2 protein levels were quantified by Western blot (WB), while h2cR and *hfq2* mRNA levels were quantified by Northern blot analysis (NB). The relative intensity of bands (RI) was estimated considering as unitary the intensity of the bands for cells of the CJ6, CJ7 or CJ8 strains grown for 2 h, and using as reference the intensity of the 5S rRNA bands. Western blot analysis was performed using 1 µg of total protein per lane, estimated in a Nanodrop ND1000 spectrophotometer. Results shown are representative of at least 4 independent experiments. Error bars are the means standard deviation.

In order to assess the levels of the Hfq2 protein, the *hfq2* mRNA, and the h2cR transcript in bacteria while infecting the nematodes, worms were harvested after 2, 8, or 24 h post-infection with the strains CJ6, CJ7 and CJ8, and processed to isolate total RNA and total protein from the bacteria thriving inside the nematodes. Results obtained indicate that the Hfq2 protein levels increased over time in the CJ6 strain (Fig. 6). The h2cR transcript levels decreased by about 5.6-fold in this strain, from maximal values detected at 2 h post-infection to minimal levels detected at 24 h, while the *hfq2* mRNA levels increased almost 2-fold over time. Consistent with the results presented in Fig. 3, the h2cR transcript in CJ6 was almost undetectable at the stationary phase. Silencing of h2cR did not change the already observed patterns of variation of the Hfq2 protein and of the *hfq2* mRNA (Fig. 6, CJ7 panel). In fact, an increase in the levels of the *hfq2* mRNA and Hfq2 protein were detected in the CJ7 strain (Fig. 6, CJ7 panel). For the strain CJ8 overexpressing the sRNA, the levels of the Hfq2 protein and of the *hfq2* mRNA showed a pattern of variation similar to that of the CJ6 strain, although both the mRNA and protein levels were highly reduced (Fig. 6, panel CJ8), except in cells grown for 24 h.

## Discussion

Recent work from our research group led to the identification of two distinct and functional Hfq-like encoding genes within the genome sequence of the opportunistic human pathogen *Burkholderia cenocepacia* J2315, as well as in other members of the *Burkholderia* genus [20], [21]. The exploitation of the ability of Hfq-like proteins to bind RNA molecules allowed us to experimentally identify sRNAs from *B. cenocepacia* J2315. In this bacterium, as well as in other members of the so-called *Burkholderia cepacia* complex (Bcc), evidences point out that several sRNAs are encoded within their genomes [29]–[31]. However, no biological functions have been attributed yet to Bcc sRNAs. Our experimental strategy allowed the identification of a sRNA, named h2cR, which is the subject of the present work. The h2cR encoding gene locates in the strand complementary to the strand encoding *hfq2*, in the opposite direction of transcription, partially overlapping the *hfq2* gene. This chromosomal location does not allow the generation of mutants on h2cR without affecting, at least, the functionality of the *hfq2* gene. Therefore, our analysis on the possible biological roles of h2cR was based on the sRNA overexpression or silencing.

Data presented indicate that h2cR is a cis-encoded negative regulator of the *hfq2* mRNA, exerting its negative regulatory effect by binding to the *hfq2* mRNA 5′-UTR region. h2cR was also found to be involved in virulence traits of *B. cenocepacia* J2315. In fact, results presented show that the sRNA affects the ability of the bacterium to persist inside the nematode's digestive tract. Previous work on the functional characterization of *hfq2* indicated a role for the encoded protein in the killing the nematodes and in their persistence in the host digestive tract [20], suggesting that the observed effects are not only due to the negative regulation exerted by h2cR on the levels of *hfq2* mRNA and, consequently, of the Hfq2 protein. Examples of sRNAs involved in the virulence of bacteria within their hosts are known. For instance, the *Streptococcus pyogenes* rivX sRNA plays an important role in virulence, linking the CovR and Mga regulatory networks, enabling the pathogen to select from its repertoire the expression of specific virulence determinants in response to a broad spectrum of environments [32]. However, no homology exists between the *B. cenocepacia* J2315 h2cR and the *S. pyogenes* rivX.

To the best of our knowledge, this is the first description of the regulation of an *hfq*-like gene by a sRNA. Interestingly, the results here presented also show that in the mutant in the other *B. cenocepacia* J2315 RNA chaperone encoding gene *hfq*, the half-life of *hfq2* mRNA was reduced compared to the wt strain, suggesting a role of the RNA chaperone Hfq on the stabilization of the *hfq2* mRNA. The role of Hfq-like proteins in the stabilization of RNA molecules has been described in other bacteria, through the protection of the RNA molecules from RNase E degradation [33]–[35]. Although there is no evidence of the occurrence of similar mechanisms in *Burkholderia* organisms, RNase E-like encoding genes are anoted in several *Burkholderia* genomes [36], [37].

Despite the numerous studies on Hfq proteins from distinct bacterial species as global regulators of bacterial metabolism and virulence, only a few studies on the regulation of expression of Hfq-like proteins encoding genes are available, none of them implicating a sRNA. These studies on the regulation of *hfq* genes are restricted to *Escherichia coli*, to the intracellular pathogen *Legionella pneumophila* and to the legume symbiotic bacterium *Sinorhizobium meliloti*. In the case of *E. coli*, the *hfq* gene is part of an operon, being its transcription complex and under the control of several promoters [38]. In this bacterium, the rate of Hfq synthesis was found as maximal in cells in the exponential growth phase, and reduced in cells in the stationary phase, being the rate of synthesis under the control of cell growth rate [39]. It has been previously shown that the *E. coli* Hfq synthesis is autoregulated at the translational level, by a mechanism involving the binding of Hfq to 2 distinct sites of the 5′-UTR region of the *hfq* mRNA, inhibiting the formation of the translation initiation complex [40]. The *S. meliloti* Hfq protein was also shown to control its own expression at the translational level, both in its natural host, as well as in the heterologous host *E. coli* [41]. In the intracellular pathogen *Legionella pneumophila*, the *hfq* gene locates in a genome region only partially resembling that of *E. coli*. For this pathogen, a regulatory scheme was proposed in which the *hfq* expression is regulated during the exponential phase of growth by the sigma factor RpoS, while during the stationary phase the gene transcription is turned off, either directly or indirectly by the regulatory protein LetA, independently of RpoS [42]. Although much remains to be known on the regulation of *hfq*-like genes in the opportunistic pathogens of the Bcc, as well as from bacteria from other genera, it is quite expectable that the involvement of a sRNA in this regulation is restricted to members of *Burkholderia* genus, since no homologs to h2cR could be found outside the *Burkholderia* genus. In addition, 2 copies of *hfq*-like genes are present only among members of the *B*. genus, *Bacillus anthracis*, *Magnetospirillum magnetotacticum* and *Novosphingobium aromaticivorans*, and in the archaeal species *Methanobacterium thermoautotrophicum* and *Archeoglobus fulgidus* [43]. Work is in progress to gain further clues on the regulation of expression of the *hfq* and *hfq2* genes, two important regulators of virulence and survival to stress conditions mimicking those faced by the opportunistic bacteria of the Bcc when infecting the cystic fibrosis host.
